# To the Editors of the Medical and Physical Journal

**Published:** 1805-07-01

**Authors:** Ed. Sutliffe

**Affiliations:** Surgeon. Bread Street Hill


### *To the Editors of the Medical and Physical Journal.*

GENTLEMEN,

USEFULNESS is the great leading motive in liberal practitioners, the accomplishment of which I conceive is exceedingly promoted by a frank disclosure of well-attested facts and cases.

Upon the subject of the Hooping Cough, medical exertions have of late been very laudably exercised, and with indisputably salutary effects; if what little I have to advance relative to the abatement or removal of that distressing malady, be approved, I shall deem the sacrifice of pecuniary remuneration more than compensated by the pleasing satisfaction afforded in having furnished medical men with what I conceive to be an acquisition for the welfare of the community, which indeed is a motive of no small magnitude to a benevolent and humane mind. It is admitted that, for the most part, medical men do not enjoy a reward equivalent either to the labor or expence incurred in their preparatory studies, or to the subsequent dangers attendant upon their professional avocations; nevertheless, I thus venture upon the candor of my medical friends, for whom I entertain an increasing esteem, and whom I consider as a society most essentially servicable in ameliorating the miseries of humanity.

The method I have been accustomed to adopt for more than ten years past, is this: R. Sulph. antim. præcipit. & spt. vini rectific partes æquales. When mixed in a copper vessel, set it on fire, stirring until the spirit is evaporated; and lest any moisture remain, I generally put it for a few minutes over a slow fire. This dried powder I give from one to five grains three or four times a day, as the age and strength of the patient, or the violence of the disease, requires. I have generally found that in two or three days it has abated the spasm and considerably promoted expectoration, and cured within a period of two weeks at the uttermost. I come far within the lines of veracity, when I assert that the successful cases, on the average, are thirty to one.

It may seem somewhat contradictory to the benevolence of the disclosure, that so great a length of time has been suffered to elapse while this method of treatment has been so little known. I readily grant the apparent reasonableness of the objection, and thus reply: Publicity by ad-

 vertisements, vertisements, generally adopted by empirics, has ever been so incompatible with my views of liberal decorum, that I could never be persuaded to employ such a method, in any modified form whatever, together with that of my union with the Royal College of Surgeons in London, since February, 1794, that I have uniformly opposed all such kind and well-meant interferences of my friends and patients, and have hence adopted the present extensive and honourable method of subscribing myself, very respectfully, &c.

*Bread Street Hill, June* 8, 1805. ED. SUTLIFFE, Surgeon.

---

